# Sensorimotor dysfunction and altered pain sensitivity in early hip osteoarthritis: associations with hip proprioception and balance impairment

**DOI:** 10.3389/fmed.2026.1873645

**Published:** 2026-06-29

**Authors:** Mastour Saeed Alshahrani, Ravi Shankar Reddy

**Affiliations:** Physical Therapy Program, Department of Medical Rehabilitation Sciences, College of Applied Medical Sciences, King Khalid University, Abha, Saudi Arabia

**Keywords:** hip osteoarthritis, pain threshold, postural balance, posturography, proprioception, sensorimotor integration

## Abstract

**Background:**

Early hip osteoarthritis is associated with subtle sensorimotor impairments and altered pain processing, which may contribute to functional limitations before advanced structural changes occur. However, the interplay between hip proprioception, postural control, and pain sensitivity remains insufficiently understood. To compare hip joint position sense between individuals with early hip osteoarthritis and asymptomatic controls, and to examine the associations among proprioception, posturographic balance, and pressure pain sensitivity.

**Methods:**

A cross-sectional study was conducted, including 38 participants with early hip osteoarthritis and 38 age- and sex-matched controls. Hip proprioception was assessed using a digital inclinometer–measured joint position sense. Postural stability was evaluated using force-platform posturography, and pressure pain thresholds were measured at local and remote sites. Between-group differences were analyzed using independent-samples *t* tests, while associations were examined using Pearson correlation and multiple linear regression analyses.

**Results:**

Participants with early hip osteoarthritis demonstrated significantly higher hip repositioning error (composite error: 5.51 ± 1.21° vs. 3.56 ± 0.92°, *p* < 0.001), higher postural sway (single-leg eyes closed COP velocity: 6.16 ± 1.51 vs. 4.64 ± 1.32 cm/s, *p* < 0.001), and lower local pressure pain thresholds (303.00 ± 68.00 vs. 415.00 ± 75.00 kPa, *p* < 0.001). Proprioceptive error was moderately associated with sway velocity (*r* = 0.56, *p* < 0.001) and inversely with local pain thresholds (*r* = −0.50, *p* = 0.001). Regression analyses indicated that proprioceptive error (*β* = 0.42, *p* = 0.004) and local pain sensitivity (*β* = −0.31, *p* = 0.021) independently predicted balance performance.

**Conclusion:**

Early hip osteoarthritis is characterized by concurrent proprioceptive deficits, balance impairments, and increased pain sensitivity, with significant interactions among these domains, suggesting that altered pain processing and sensorimotor dysfunction are interconnected features of early hip osteoarthritis.

## 1 Introduction

Hip osteoarthritis is a prevalent musculoskeletal condition and a major contributor to pain, reduced mobility, and functional limitation in middle-aged and older adults (1). Although traditionally considered a disease of advanced structural degeneration, increasing attention has been directed toward early-stage hip osteoarthritis, where clinical symptoms emerge despite relatively mild radiographic changes (2). Functional impairments in this stage are often multifactorial, involving not only joint morphology but also neuromuscular and sensorimotor alterations (3). Proprioception, defined as the ability to perceive joint position and movement, plays a critical role in maintaining joint stability and coordinated movement, while postural control depends on the integration of proprioceptive, visual, and vestibular inputs (4–6). Additionally, alterations in pain processing, including reduced pressure pain thresholds, may influence both sensory input and motor output, thereby contributing to functional deficits (7).

Previous research has demonstrated that individuals with lower-limb osteoarthritis exhibit impairments in joint position sense and postural stability, particularly under conditions of increased sensory demand (5, 8, 9). Studies in populations with knee and hip osteoarthritis have reported lower proprioceptive acuity and higher postural sway, suggesting compromised sensorimotor control (10, 11). Furthermore, emerging evidence indicates that pain sensitivity, including both peripheral and central components, is altered in osteoarthritis and may be associated with functional performance (12, 13). Investigations have shown that reduced pressure pain thresholds are present not only locally but also at remote sites, reflecting broader changes in nociceptive processing (14, 15). However, most existing studies have examined these domains independently, with limited integration of proprioception, balance, and pain sensitivity within the same cohort, particularly in early hip osteoarthritis (16).

A comprehensive understanding of how these factors interact in the early stages of disease is clinically important, as early identification of modifiable impairments may inform targeted rehabilitation strategies (17). While prior studies have identified proprioceptive deficits and balance impairments in osteoarthritis, the extent to which these are associated with altered pain sensitivity remains insufficiently characterized (18). Moreover, the relative contributions of proprioceptive error and pain sensitivity to postural instability have not been clearly established using objective, clinically applicable measures (18). Addressing these gaps may provide insight into the mechanisms underlying functional decline and support the development of integrated assessment approaches in early hip osteoarthritis (18).

The present study aimed to compare hip joint position sense, posturographic balance performance, and pressure pain thresholds between individuals with early hip osteoarthritis and asymptomatic controls, and to examine the associations among proprioceptive accuracy, postural instability, and pain sensitivity in the early hip osteoarthritis group. It was hypothesized that individuals with early-stage hip osteoarthritis would exhibit greater proprioceptive error and postural instability, along with reduced pressure pain thresholds, compared with controls. It was further hypothesized that higher proprioceptive deficits would be associated with higher postural sway and lower pressure pain thresholds, and that proprioceptive error and pain sensitivity would independently contribute to balance impairment.

## 2 Methods

### 2.1 Study design, ethics, and settings

This cross-sectional study was conducted between January 2024 and December 2024 at the Physiotherapy Clinic, Department of Rehabilitation Sciences, King Khalid University, Kingdom of Saudi Arabia. Ethical approval was obtained from the Institutional Review Board of King Khalid University (REC# 456–2024), and written informed consent was obtained from all participants prior to inclusion. The study was carried out in accordance with the ethical principles outlined in the Declaration of Helsinki.

### 2.2 Sample size calculation

Sample size was calculated using G*Power software (version 3.1.9.7) for the primary comparison of hip joint position-sense error between individuals with early hip osteoarthritis and asymptomatic controls. The calculation was based on an independent-samples *t*-test with two groups, a two-tailed alpha level of 0.05, statistical power of 0.80, and an allocation ratio of 1:1. An expected between-group effect size of Cohen’s d = 0.65 was assumed based on previously reported differences in hip joint reposition sense in unilateral hip osteoarthritis, where hip repositioning error was higher in the osteoarthritis group than in controls for flexion (4.96 ± 1.09° vs. 2.45 ± 0.23°) and abduction (5.67 ± 1.88° vs. 2.56 ± 0.98°) (5). The analysis indicated that 38 participants were required per group, resulting in a total sample size of 76 participants.

### 2.3 Participants

Participants with early hip osteoarthritis were recruited from outpatient physiotherapy and orthopedic clinics via consecutive sampling and clinician referrals. Asymptomatic controls were recruited from the university community through advertisements and word of mouth. Eligibility screening included a medical history review, a physical examination, and radiographic verification, all conducted by a qualified physician prior to enrollment. Inclusion criteria for the early hip osteoarthritis group comprised adults aged 40–70 years with unilateral or bilateral hip symptoms, hip or groin pain for at least 3 months, radiographic evidence corresponding to Kellgren–Lawrence grade 1 or 2 on standard anteroposterior pelvic radiographs (13, 14), and the ability to ambulate independently. Control participants were required to have no history of hip pain, musculoskeletal disorders, or lower-limb functional limitations. Exclusion criteria for both groups included prior hip surgery, advanced hip osteoarthritis (Kellgren–Lawrence grade ≥3), neurological or vestibular disorders, systemic inflammatory conditions, recent lower-limb injury, or any condition that could influence balance or proprioception. Following confirmation of eligibility, baseline assessments were conducted for all participants prior to data collection. For participants with unilateral symptoms, the affected limb was assessed. In cases of bilateral symptoms, the more symptomatic limb, as determined by self-reported pain intensity, was selected for analysis. For control participants, the dominant limb was assessed.

### 2.4 Hip joint position sense

Hip joint position sense (JPS) was defined as the ability to actively reproduce a predetermined joint angle (19, 20). The primary proprioceptive outcome measure was the composite absolute repositioning error, calculated as the mean of the absolute repositioning errors across hip flexion, abduction, internal rotation, and external rotation directions (19). It was assessed using a calibrated digital inclinometer (JTECH Medical, Midvale, UT, USA), which has demonstrated acceptable reliability and validity for lower-limb proprioception assessment in musculoskeletal populations (19). Participants were tested in standardized positions depending on the movement direction: supine for hip flexion, side-lying for abduction, and sitting for internal and external rotation (19). A target angle (30° flexion, 20° abduction, and 15° internal and external rotation) was passively demonstrated by the examiner, after which the limb was returned to the starting position (19). Participants were then instructed to actively reproduce the target angle without visual feedback. Three trials were performed for each movement, with a rest interval of approximately 30 s between trials to minimize fatigue. The digital inclinometer was aligned with anatomical landmarks (the lateral femoral condyle and greater trochanter) to ensure consistent measurements. Absolute repositioning error (difference between target and reproduced angle), constant error (directional bias), and variable error (standard deviation of repeated trials) were calculated in degrees (16). Although multiple JPS-derived variables were analyzed to comprehensively characterize proprioceptive performance, the composite absolute repositioning error was considered the primary proprioceptive outcome measure. To enhance measurement reliability, all assessments were performed by the same trained examiner with prior experience in musculoskeletal evaluation. A standardized protocol was followed for all measurements. Pilot testing was conducted prior to data collection to ensure intra-rater consistency. The assessor was not blinded to group allocation due to the nature of the study; however, objective measurement tools were used to minimize potential bias.

### 2.5 Posturographic balance assessment

**Figure 1:**
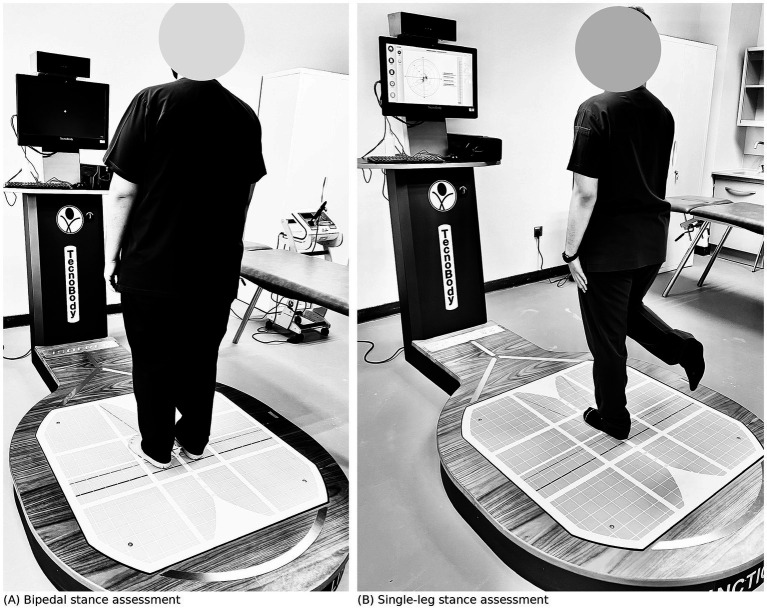
Force-platform posturographic assessment procedures during bipedal and single-leg stance conditions in participants with early hip osteoarthritis. **(A)** Bipedal stance assessment performed on the Iso-Free force-platform posturography system under standardized testing conditions. **(B)** Single-leg stance assessment is used to evaluate postural stability and center-of-pressure displacement during increased balance demand.

Postural stability was assessed using a force-platform–based posturography system (iso-free, Technobody S.r.l., Bergamo, Italy), which provides objective quantification of center-of-pressure (COP) displacement (21). Participants performed balance tasks under standardized conditions comprising bipedal stance with eyes open, bipedal stance with eyes closed, single-leg stance with eyes open, and single-leg stance with eyes closed (Figure 1). The order of testing conditions was standardized for all participants to ensure consistency and minimize variability related to fatigue or learning effects. Each trial lasted 30 s, and three trials were recorded per condition, with adequate rest intervals between trials. Participants were instructed to stand as still as possible with arms by their sides, and foot placement was standardized using predefined markers on the platform. The primary posturographic variables extracted were COP path length (cm), mean COP velocity (cm/s), sway area (95% confidence ellipse, cm^2^), and anteroposterior and mediolateral sway ranges (cm) (21). Data were sampled at 100 Hz using the Iso-Free force platform (Technobody S.r.l., Bergamo, Italy) and processed with the manufacturer’s proprietary software (22). The average of the three trials for each condition was used for analysis. A composite postural instability score was calculated by standardizing (z-scoring) selected posturographic variables (COP path length, velocity, and sway area across conditions) and averaging them to provide an overall index of balance performance. Higher values indicated higher instability. Increased COP displacement and velocity were interpreted as indicators of impaired postural control. The testing protocol and outcome measures align with established guidelines for posturographic assessment in clinical populations.

### 2.6 Pressure pain threshold

Pressure pain threshold (PPT) was defined as the minimum pressure required to elicit the first sensation of pain and was measured with a handheld digital pressure algometer with a 1 cm^2^ probe area (Baseline® Push-Pull Force Gauge, Fabrication Enterprises Inc., White Plains, NY, USA) (23, 24). PPT assessments were conducted at standardized anatomical sites, including local hip-related regions (anterior hip joint line, greater trochanter, adductor region, iliopsoas, and gluteus medius) and remote sites (knee, tibialis anterior, and contralateral forearm) (24). The algometer was applied perpendicular to the skin at a constant rate of approximately 30 kPa/s, as recommended in prior studies. Participants were instructed to indicate the point at which pressure first became painful, at which time the reading was recorded in kilopascals (kPa) (24). Three measurements were obtained at each site, with a 30–60-s rest interval between measurements, and the mean value was used for analysis. Composite PPT values for local and remote regions were calculated by averaging respective sites. The local-to-remote PPT ratio was calculated by dividing the mean local PPT by the mean remote PPT. Side-to-side asymmetry was calculated as the percentage difference between limbs using the formula: [(higher value − lower value) / higher value × 100] (25). Lower PPT values indicated increased pain sensitivity. The methodology followed standardized procedures widely used in musculoskeletal pain research.

### 2.7 Demographic and anthropometric variables

Demographic and anthropometric variables comprised age, sex, height, weight, and body mass index (BMI), which were collected to characterize the sample and control for potential confounding effects. Height was measured using a stadiometer to the nearest 0.1 cm, and body weight was measured using a calibrated digital scale to the nearest 0.1 kg. BMI was calculated as weight in kilograms divided by height in meters squared (kg/m^2^). Additional clinical variables included physical activity level, assessed as self-reported minutes per week of moderate-to-vigorous activity using a standardized questionnaire adapted from the International Physical Activity Questionnaire (IPAQ), and pain intensity, measured on an 11-point numeric rating scale (0–10), with higher scores indicating greater pain. These variables were included in regression models as potential covariates due to their known influence on balance, proprioception, and pain perception.

### 2.8 Radiographic and clinical characteristics

Radiographic severity of hip osteoarthritis was classified using the Kellgren–Lawrence grading system based on standard pelvic radiographs, with grades 1 and 2 indicating early disease (26). Clinical characteristics comprised symptom duration (months), symptom laterality (unilateral or bilateral), and medication use, particularly regular use of analgesics or non-steroidal anti-inflammatory drugs. Functional performance measures, including the Timed Up and Go test (27), 30-s chair-stand test (28), and six-minute walk test (29), were recorded using standardized protocols to provide additional context regarding physical function. Hip range of motion (ROM) was assessed using a standard universal goniometer in accordance with established clinical procedures. Measurements included hip flexion, abduction, internal rotation, and external rotation, recorded in degrees. Three trials were performed for each movement, and the mean value was used for analysis. The TUG test was conducted by instructing participants to rise from a chair, walk 3 meters, turn, walk back, and sit down; the time (in seconds) was recorded (27). The 30-s chair-stand test assessed lower-limb functional strength by counting the number of full stands completed in 30 s (28). The six-minute walk test (6MWT) measured submaximal functional capacity as the total distance walked (meters) over 6 minutes along a standardized walkway (29). Waist circumference was measured at the midpoint between the lowest rib and the iliac crest using a non-elastic tape. Resting blood pressure was measured using an automated sphygmomanometer after 5 min of seated rest, with the average of two readings recorded. Although not primary outcomes, these variables were included to comprehensively characterize the study population and to ensure consistency with the existing literature on early hip osteoarthritis. All procedures followed standardized clinical guidelines to ensure reliability and reproducibility.

### 2.9 Statistical analysis

Data were analyzed using IBM SPSS Statistics for Windows, version 24.0 (IBM Corp., Armonk, NY, USA). Normality of data distribution was confirmed using the Shapiro–Wilk test and visual inspection of histograms and Q–Q plots. Descriptive statistics were calculated and presented as mean ± standard deviation for continuous variables and frequencies with percentages for categorical variables. Between-group comparisons (early hip osteoarthritis vs. controls) for continuous variables, including hip joint position sense errors, posturographic parameters, and pressure pain thresholds, were performed using independent-samples *t* tests, while categorical variables were analyzed using chi-square tests. For Objective 2, associations among proprioception, balance, and pain sensitivity variables within the early hip osteoarthritis group were examined using Pearson correlation coefficients with corresponding 95% confidence intervals. Subsequently, two multiple linear regression models were constructed using the enter method to identify independent predictors of posturographic balance impairment and hip proprioceptive error. In Model 1, center-of-pressure sway velocity during single-leg stance with eyes closed was entered as the dependent variable, whereas composite hip joint position-sense absolute error, local anterior hip pressure pain threshold, age, body mass index, and pain intensity were entered simultaneously as predictor variables. In Model 2, composite hip joint position-sense absolute error was entered as the dependent variable, while center-of-pressure sway velocity during single-leg stance with eyes closed, local anterior hip pressure pain threshold, age, body mass index, and pain intensity were entered simultaneously as predictor variables. Age, body mass index, and pain intensity were included as adjustment covariates because of their known influence on sensorimotor function, postural control, and pain perception in musculoskeletal populations. Independent variables included in regression models were selected based on theoretical relevance and significant associations observed in correlation analyses. All selected predictors were entered simultaneously using the enter method. Pressure pain threshold variables were scaled per 100 kPa to facilitate interpretation of regression coefficients. The number of predictors included in regression models was limited in accordance with recommended subject-to-variable ratios to reduce the risk of model overfitting. Assumptions of linearity, independence, homoscedasticity, and absence of multicollinearity were verified prior to model interpretation. A *p*-value of <0.05 was considered statistically significant for all analyses. No formal adjustment for multiple comparisons was applied because the study was exploratory; therefore, statistically significant findings were interpreted with consideration of the potential for increased Type I error.

## 3 Results

Compared with controls, participants with early hip osteoarthritis demonstrated significantly higher BMI (28.82 ± 3.71 vs. 26.94 ± 3.18; *p* = 0.020), lower physical activity levels (118.42 ± 55.31 vs. 152.89 ± 61.84 min/week; *p* = 0.012), higher pain intensity (4.82 ± 1.41 vs. 0.32 ± 0.53; *p* < 0.001), and lower hip range of motion, particularly internal rotation (24.68 ± 7.15 vs. 35.76 ± 8.02; *p* < 0.001). Functional performance measures were lower in the early hip osteoarthritis group, as reflected by slower Timed Up and Go performance (8.42 ± 1.18 vs. 7.36 ± 0.94 s; *p* < 0.001), fewer chair-stand repetitions (11.84 ± 2.76 vs. 14.61 ± 3.12; *p* < 0.001), and a lower six-minute walk distance by approximately 64 m (*p* < 0.001). Additionally, analgesic use was substantially higher in the early hip OA group (36.84% vs. 2.63%; *p* < 0.001), indicating clinically meaningful functional impairment and symptom burden (Table 1).

**Table 1:** Demographic, clinical, radiographic, and functional characteristics of participants with early hip osteoarthritis and asymptomatic controls (*n* = 76).

Variable	Early hip OA (*n* = 38)	Control (*n* = 38)	95% CI for mean difference	*p*-value
Age, years	58.76 ± 6.47	57.92 ± 6.31	−2.08 to 3.76	0.568
Height, cm	166.84 ± 8.21	168.23 ± 7.94	−5.08 to 2.30	0.456
Weight, kg	80.32 ± 12.76	76.41 ± 11.83	−1.71 to 9.53	0.170
BMI, kg/m^2^	28.82 ± 3.71	26.94 ± 3.18	0.30 to 3.46	0.020
Waist circumference, cm	96.18 ± 10.42	91.76 ± 9.87	−0.22 to 9.06	0.062
Resting systolic BP, mmHg	127.34 ± 12.15	123.08 ± 11.42	−1.13 to 9.65	0.120
Resting diastolic BP, mmHg	78.29 ± 8.62	76.18 ± 7.94	−1.68 to 5.90	0.271
Physical activity, min/week	118.42 ± 55.31	152.89 ± 61.84	−61.29 to −7.65	0.012
Sex, female	22 (57.89)	21 (55.26)	—	0.817
Dominant/assessed side, right	21 (55.26)	19 (50.00)	—	0.646
Current smoker	5 (13.16)	4 (10.53)	—	0.723
At least one cardiometabolic comorbidity	12 (31.58)	8 (21.05)	—	0.297
Symptom duration, months	18.63 ± 8.74	—	—	—
Unilateral hip symptoms	26 (68.42)	—	—	—
KL grade 1	17 (44.74)	—	—	—
KL grade 2	21 (55.26)	—	—	—
Regular analgesic/NSAID use	14 (36.84)	1 (2.63)	—	<0.001
Pain intensity, 0–10 NRS	4.82 ± 1.41	0.32 ± 0.53	4.01 to 4.99	<0.001
Affected/dominant hip flexion ROM, °	105.34 ± 9.72	116.18 ± 8.53	−15.02 to −6.66	<0.001
Hip abduction ROM, °	35.71 ± 6.83	43.29 ± 7.36	−10.83 to −4.33	<0.001
Hip internal rotation ROM, °	24.68 ± 7.15	35.76 ± 8.02	−14.55 to −7.61	<0.001
Hip external rotation ROM, °	33.42 ± 8.24	40.58 ± 7.91	−10.85 to −3.47	<0.001
Timed Up and Go, s	8.42 ± 1.18	7.36 ± 0.94	0.57 to 1.55	<0.001
30-s chair-stand test, repetitions	11.84 ± 2.76	14.61 ± 3.12	−4.12 to −1.42	<0.001
Six-minute walk distance, m	502.63 ± 68.42	566.79 ± 72.88	−96.47 to −31.85	<0.001

Values are presented as mean ± standard deviation or number (percentage). Mean differences were calculated as early hip OA minus control. Continuous variables were compared using independent-samples *t* tests; categorical variables were compared using chi-square tests; variables measured only in the early hip OA group were summarized descriptively. BMI, body mass index; BP, blood pressure; CI, confidence interval; KL, Kellgren-Lawrence; NRS, numerical rating scale; NSAID, non-steroidal anti-inflammatory drug; OA, osteoarthritis; ROM, range of motion.

Hip proprioception was consistently impaired in early hip osteoarthritis, with significantly higher absolute repositioning errors across all movements, most notably internal rotation (6.14 ± 1.62 vs. 3.96 ± 1.31; mean difference 2.18°, *p* < 0.001) and a higher composite error (5.51 ± 1.21 vs. 3.56 ± 0.92; *p* < 0.001) (Table 2). Variable error was also elevated, indicating reduced consistency, particularly for internal rotation (2.54 ± 0.89 vs. 1.73 ± 0.68; *p* < 0.001).

**Table 2:** Digital-inclinometer-measured hip proprioception outcomes in participants with early hip osteoarthritis and asymptomatic controls.

Hip motion	Target angle	Error type	Early hip OA group (*n* = 38)	Control group (*n* = 38)	Mean difference (OA-Control)	95% CI	*p*-value
Flexion	30°	Absolute error	4.82 ± 1.36	3.12 ± 1.05	1.70	1.14 to 2.26	<0.001
		Constant error	1.28 ± 1.12	0.42 ± 0.88	0.86	0.40 to 1.32	<0.001
		Variable error	2.04 ± 0.74	1.37 ± 0.55	0.67	0.37 to 0.97	<0.001
Abduction	20°	Absolute error	5.36 ± 1.48	3.48 ± 1.18	1.88	1.27 to 2.49	<0.001
		Constant error	1.52 ± 1.20	0.51 ± 0.91	1.01	0.52 to 1.50	<0.001
		Variable error	2.26 ± 0.81	1.52 ± 0.61	0.74	0.41 to 1.07	<0.001
Internal rotation	15°	Absolute error	6.14 ± 1.62	3.96 ± 1.31	2.18	1.51 to 2.85	<0.001
		Constant error	1.76 ± 1.29	0.64 ± 0.98	1.12	0.60 to 1.64	<0.001
		Variable error	2.54 ± 0.89	1.73 ± 0.68	0.81	0.45 to 1.17	<0.001
External rotation	15°	Absolute error	5.71 ± 1.55	3.68 ± 1.23	2.03	1.39 to 2.67	<0.001
		Constant error	1.63 ± 1.24	0.57 ± 0.95	1.06	0.55 to 1.57	<0.001
		Variable error	2.39 ± 0.84	1.61 ± 0.63	0.78	0.44 to 1.12	<0.001
Composite	Average of four directions	Absolute error	5.51 ± 1.21	3.56 ± 0.92	1.95	1.46 to 2.44	<0.001
Composite	Average of four directions	Variable error	2.31 ± 0.68	1.56 ± 0.48	0.75	0.48 to 1.02	<0.001

Absolute repositioning error represented the unsigned difference between the target and reproduced hip angle; constant error represented directional bias; variable error represented trial-to-trial inconsistency. Composite scores were calculated as the arithmetic mean of flexion, abduction, internal rotation, and external rotation outcomes. Values are presented as mean ± standard deviation unless otherwise indicated. Between-group comparisons were performed using independent-samples *t* tests.

CI, confidence interval; OA, osteoarthritis; SD, standard deviation.

Posturographic analysis demonstrated significantly higher postural instability in early hip osteoarthritis across all conditions, with the largest differences observed during single-leg stance with eyes closed, including COP path length (184.76 ± 45.38 vs. 139.24 ± 39.56 cm; *p* < 0.001) and sway velocity (6.16 ± 1.51 vs. 4.64 ± 1.32 cm/s; *p* < 0.001) (Table 3). Sway area was also markedly increased (18.92 ± 5.46 vs. 13.51 ± 4.72 cm^2^; *p* < 0.001), indicating impaired balance control under challenging conditions. Even under bipedal and eyes-open conditions, significant differences persisted, and composite instability scores were higher in OA (0.62 ± 0.71 vs. − 0.02 ± 0.65; *p* < 0.001).

**Table 3:** Posturographic balance outcomes in individuals with early hip osteoarthritis and asymptomatic controls.

Balance domain	Stance condition	Vision	Unit	Early hip OA (*n* = 38)	Control (*n* = 38)	Mean difference (95% CI)	*p*-value
COP path length	Bipedal	Eyes open	cm	42.85 ± 11.62	35.14 ± 9.48	7.71 (2.86 to 12.56)	0.002
COP path length	Bipedal	Eyes closed	cm	58.36 ± 15.27	45.72 ± 12.44	12.64 (6.27 to 19.01)	<0.001
COP path length	Single-leg	Eyes open	cm	115.42 ± 28.65	89.38 ± 24.17	26.04 (13.92 to 38.16)	<0.001
COP path length	Single-leg	Eyes closed	cm	184.76 ± 45.38	139.24 ± 39.56	45.52 (26.05 to 64.99)	<0.001
Mean COP velocity	Bipedal	Eyes open	cm/s	1.43 ± 0.39	1.17 ± 0.32	0.26 (0.10 to 0.42)	0.002
Mean COP velocity	Bipedal	Eyes closed	cm/s	1.95 ± 0.51	1.53 ± 0.42	0.42 (0.21 to 0.63)	<0.001
Mean COP velocity	Single-leg	Eyes open	cm/s	3.85 ± 0.96	2.98 ± 0.81	0.87 (0.46 to 1.28)	<0.001
Mean COP velocity	Single-leg	Eyes closed	cm/s	6.16 ± 1.51	4.64 ± 1.32	1.52 (0.87 to 2.17)	<0.001
95% confidence ellipse sway area	Bipedal	Eyes open	cm^2^	2.84 ± 1.08	2.05 ± 0.86	0.79 (0.34 to 1.24)	<0.001
95% confidence ellipse sway area	Bipedal	Eyes closed	cm^2^	4.62 ± 1.69	3.24 ± 1.31	1.38 (0.69 to 2.07)	<0.001
95% confidence ellipse sway area	Single-leg	Eyes open	cm^2^	9.76 ± 3.18	6.84 ± 2.56	2.92 (1.60 to 4.24)	<0.001
95% confidence ellipse sway area	Single-leg	Eyes closed	cm^2^	18.92 ± 5.46	13.51 ± 4.72	5.41 (3.08 to 7.74)	<0.001
AP sway range	Bipedal	Eyes open	cm	2.18 ± 0.72	1.74 ± 0.61	0.44 (0.13 to 0.75)	0.005
AP sway range	Bipedal	Eyes closed	cm	3.06 ± 0.94	2.37 ± 0.83	0.69 (0.28 to 1.10)	0.001
ML sway range	Bipedal	Eyes open	cm	1.86 ± 0.64	1.42 ± 0.53	0.44 (0.17 to 0.71)	0.002
ML sway range	Bipedal	Eyes closed	cm	2.74 ± 0.86	2.03 ± 0.76	0.71 (0.34 to 1.08)	<0.001
AP sway range	Single-leg	Eyes open	cm	5.84 ± 1.52	4.36 ± 1.31	1.48 (0.83 to 2.13)	<0.001
ML sway range	Single-leg	Eyes open	cm	4.95 ± 1.37	3.58 ± 1.16	1.37 (0.79 to 1.95)	<0.001
Composite balance	Overall	—	z-score	0.62 ± 0.71	−0.02 ± 0.65	0.64 (0.33 to 0.95)	<0.001

Between-group comparisons were performed using independent-samples *t* tests, with mean differences and 95% confidence intervals reported for early hip osteoarthritis minus control values. Values are presented as mean ± standard deviation unless otherwise indicated.

AP, anteroposterior; CI, confidence interval; COP, center of pressure; ML, mediolateral; OA, osteoarthritis; SD, standard deviation.

Pressure pain thresholds were significantly lower in the early hip osteoarthritis group at both local and remote sites, with the largest reductions observed at the greater trochanter (302.00 ± 76.00 vs. 418.00 ± 86.00 kPa; *p* < 0.001, d = −1.43) and anterior hip (285.00 ± 72.00 vs. 392.00 ± 83.00 kPa; *p* < 0.001) (Table 4). The composite local PPT was reduced by 112.00 kPa (*p* < 0.001), indicating pronounced peripheral sensitization. Remote PPTs were also lower, though with smaller effect sizes (e.g., forearm: *p* = 0.034), suggesting elements of widespread sensitivity. Additionally, the local-to-remote PPT ratio was significantly decreased (0.72 ± 0.13 vs. 0.87 ± 0.14; *p* < 0.001), and side-to-side asymmetry was higher (16.80 ± 7.40% vs. 8.90 ± 5.10%; *p* < 0.001), reinforcing altered pain processing.

**Table 4:** Pressure pain threshold outcomes at local hip-related and remote reference sites in participants with early hip osteoarthritis and asymptomatic controls.

Outcome	Early hip OA (*n* = 38) Mean ± SD	Control (*n* = 38) Mean ± SD	Mean difference (95% CI)	*t* value	*p* value	Cohen’s d
Anterior hip joint line PPT, kPa	285.00 ± 72.00	392.00 ± 83.00	−107.00 (−142.52 to −71.48)	−6.00	<0.001	−1.38
Greater trochanter PPT, kPa	302.00 ± 76.00	418.00 ± 86.00	−116.00 (−153.10 to −78.90)	−6.23	<0.001	−1.43
Adductor longus region PPT, kPa	318.00 ± 81.00	436.00 ± 91.00	−118.00 (−157.38 to −78.62)	−5.97	<0.001	−1.37
Iliopsoas region PPT, kPa	276.00 ± 70.00	381.00 ± 79.00	−105.00 (−139.12 to −70.88)	−6.13	<0.001	−1.41
Gluteus medius region PPT, kPa	334.00 ± 85.00	447.00 ± 94.00	−113.00 (−153.96 to −72.04)	−5.50	<0.001	−1.26
Local hip PPT composite, kPa	303.00 ± 68.00	415.00 ± 75.00	−112.00 (−144.72 to −79.28)	−6.82	<0.001	−1.56
Knee remote PPT, kPa	421.00 ± 96.00	485.00 ± 105.00	−64.00 (−109.99 to −18.01)	−2.77	0.007	−0.64
Tibialis anterior remote PPT, kPa	463.00 ± 104.00	535.00 ± 118.00	−72.00 (−122.84 to −21.16)	−2.82	0.006	−0.65
Contralateral forearm remote PPT, kPa	376.00 ± 88.00	421.00 ± 94.00	−45.00 (−86.62 to −3.38)	−2.15	0.034	−0.49
Remote PPT composite, kPa	420.00 ± 82.00	480.00 ± 88.00	−60.00 (−98.88 to −21.12)	−3.07	0.003	−0.71
Local-to-remote PPT ratio	0.72 ± 0.13	0.87 ± 0.14	−0.15 (−0.21 to −0.09)	−4.84	<0.001	−1.11
Side-to-side PPT asymmetry, %	16.80 ± 7.40	8.90 ± 5.10	7.90 (5.00 to 10.80)	5.42	<0.001	1.24

Between-group comparisons were performed using independent-samples *t* tests. Mean difference represents early hip OA minus control values; negative values indicate lower pressure pain thresholds in the early hip OA group.

CI, confidence interval; kPa, kilopascal; OA, osteoarthritis; PPT, pressure pain threshold; SD, standard deviation.

**Figure 2:**
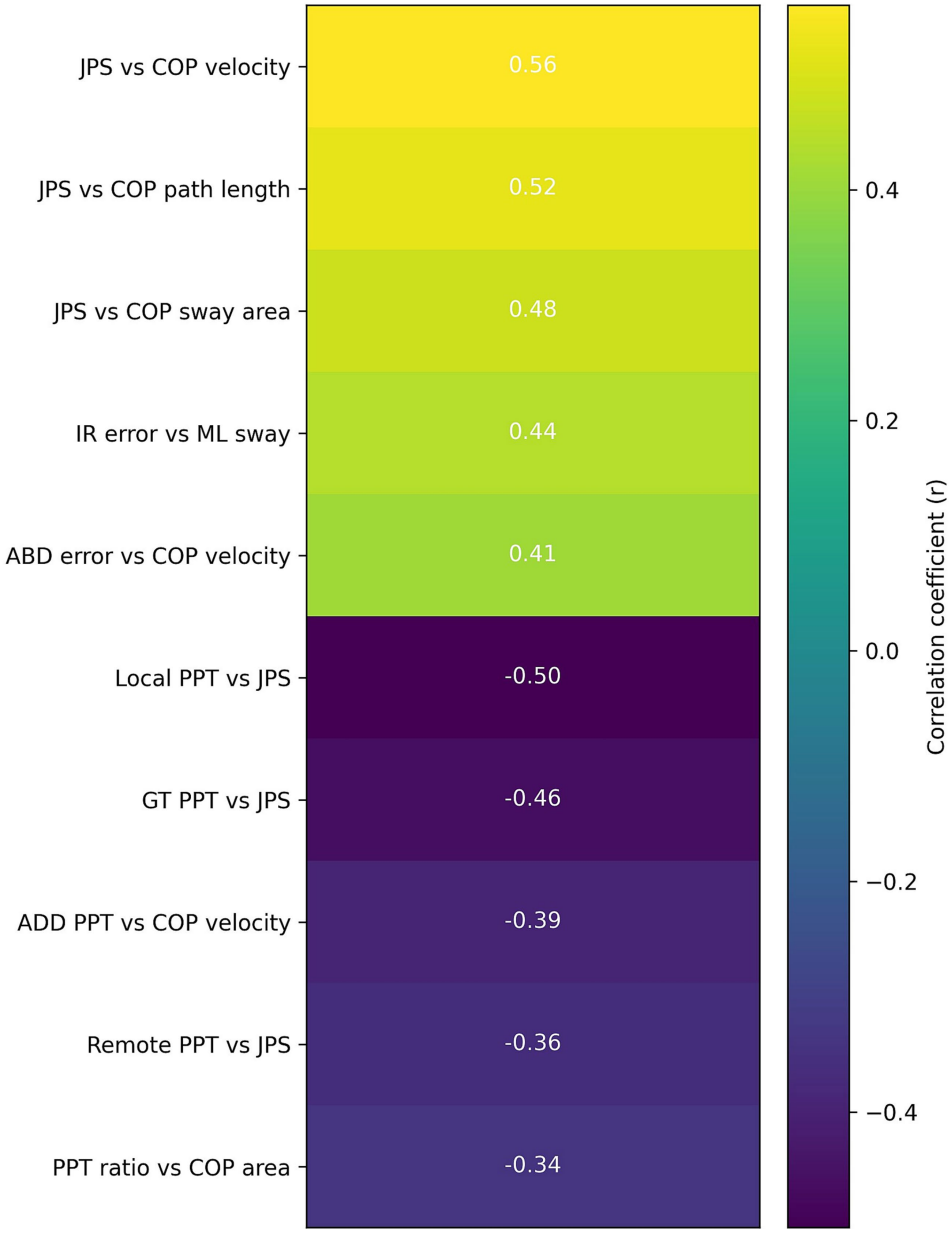
Heatmap of Pearson correlation coefficients between hip proprioception, Posturographic balance parameters, and pressure pain sensitivity in early hip osteoarthritis.

Moderate positive correlations were identified between hip proprioceptive error and posturographic instability, particularly for COP sway velocity (*r* = 0.56, *p* < 0.001) and path length (*r* = 0.52, *p* < 0.001) under eyes-closed conditions (Table 5; Figure 2). Proprioceptive deficits were also moderately associated with pain sensitivity, as indicated by inverse correlations with local PPT (*r* = −0.50, *p* = 0.001) and greater trochanter PPT (*r* = −0.46, *p* = 0.004). Weaker associations were observed with remote PPT (*r* = −0.36, *p* = 0.026), suggesting a gradient of sensory involvement. Additionally, lower PPT ratios were associated with higher sway area (*r* = −0.34, *p* = 0.037), indicating an interaction between peripheral sensitivity and balance control.

**Table 5:** Pearson correlation analysis of associations among digital inclinometer–measured hip joint position sense, posturographic balance parameters, and pressure pain thresholds in individuals with early hip osteoarthritis (*n* = 38).

Association	Predictor	Outcome	*r*	95% CI for *r*	*p*-value
1	Composite hip JPS absolute error (°)	COP sway velocity, single-leg stance, eyes closed (cm/s)	0.56	0.29 to 0.75	<0.001
2	Composite hip JPS absolute error (°)	COP path length, eyes closed (cm)	0.52	0.24 to 0.72	<0.001
3	Composite hip JPS absolute error (°)	COP sway area, eyes closed (cm^2^)	0.48	0.19 to 0.69	0.002
4	Hip internal rotation absolute error (°)	Mediolateral sway, eyes closed (cm)	0.44	0.14 to 0.67	0.006
5	Hip abduction absolute error (°)	Single-leg stance COP velocity, eyes open (cm/s)	0.41	0.10 to 0.65	0.011
6	Local anterior hip PPT (kPa)	Composite hip JPS absolute error (°)	−0.50	−0.71 to −0.21	0.001
7	Greater trochanter PPT (kPa)	Composite hip JPS absolute error (°)	−0.46	−0.68 to −0.16	0.004
8	Adductor-region PPT (kPa)	COP sway velocity, single-leg stance, eyes closed (cm/s)	−0.39	−0.63 to −0.08	0.015
9	Remote forearm PPT (kPa)	Composite hip JPS absolute error (°)	−0.36	−0.61 to −0.05	0.026
10	Local-to-remote PPT ratio	COP sway area, eyes closed (cm^2^)	−0.34	−0.60 to −0.02	0.037

Pearson correlation coefficients (*r*), with corresponding 95% confidence intervals (CIs) and *p*-values, are presented for associations among hip joint position sense (JPS) errors, posturographic balance variables, and pressure pain threshold (PPT) measures. All analyses were conducted within the early hip osteoarthritis group only (*n* = 38). Positive *r* values indicate that higher proprioceptive error is associated with higher postural sway, whereas negative *r* values indicate that lower PPT (higher pain sensitivity) is associated with higher proprioceptive error or impaired balance. Statistical significance was set at *p* < 0.05.

CI, confidence interval; COP, center of pressure; JPS, joint position sense; PPT, pressure pain threshold; *r*, Pearson correlation coefficient.

**Figure 3:**
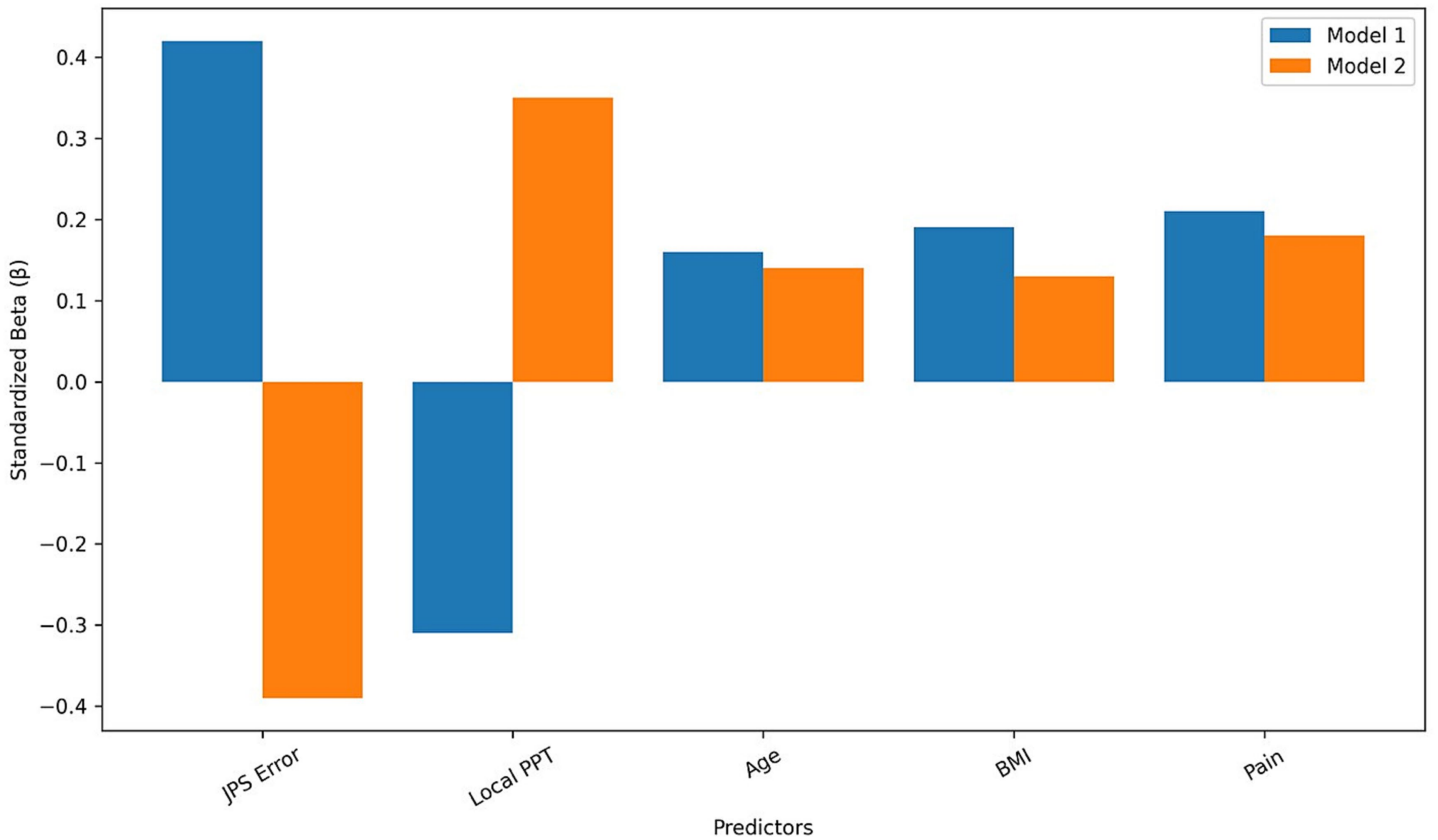
Standardized regression coefficients (*β*) for predictors of posturographic balance and hip proprioceptive error in early hip osteoarthritis.

Regression analyses identified hip proprioceptive error (*β* = 0.42, *p* = 0.004) and local PPT (*β* = −0.31, *p* = 0.021) as independent predictors of postural sway velocity, explaining 38% of variance (adjusted R^2^ = 0.30) (Table 6; Figure 3). In the second model, lower local PPT (*β* = −0.39, *p* = 0.002) and higher sway velocity (*β* = 0.35, *p* = 0.006) were significant predictors of higher proprioceptive error, with similar explanatory power (adjusted R^2^ = 0.28).

**Table 6:** Multiple linear regression models examining determinants of posturographic balance and hip proprioceptive error in individuals with early hip osteoarthritis (*n* = 38).

Model	Dependent variable	Independent variable	B	SE	*β*	95% CI for B	*p*-value	Model R^2^/adjusted R^2^
Model 1	COP sway velocity, single-leg stance, eyes closed (cm/s)	Composite hip JPS absolute error (°)	0.09	0.03	0.42	0.03 to 0.15	0.004	0.38/0.30
Model 1	COP sway velocity, single-leg stance, eyes closed (cm/s)	Local anterior hip PPT (per 100 kPa)	−0.07	0.03	−0.31	−0.13 to −0.01	0.021	
Model 1	COP sway velocity, single-leg stance, eyes closed (cm/s)	Age (years)	0.01	0.01	0.16	−0.01 to 0.03	0.197	
Model 1	COP sway velocity, single-leg stance, eyes closed (cm/s)	BMI (kg/m^2^)	0.02	0.01	0.19	−0.01 to 0.05	0.118	
Model 1	COP sway velocity, single-leg stance, eyes closed (cm/s)	Pain intensity (0–10 NRS)	0.04	0.03	0.21	−0.02 to 0.10	0.142	
Model 2	Composite hip JPS absolute error (°)	Local anterior hip PPT (per 100 kPa)	−0.42	0.13	−0.39	−0.68 to −0.16	0.002	0.36/0.28
Model 2	Composite hip JPS absolute error (°)	COP sway velocity, single-leg stance, eyes closed (cm/s)	1.24	0.43	0.35	0.37 to 2.11	0.006	
Model 2	Composite hip JPS absolute error (°)	Age (years)	0.03	0.03	0.14	−0.03 to 0.09	0.273	
Model 2	Composite hip JPS absolute error (°)	BMI (kg/m^2^)	0.05	0.05	0.13	−0.05 to 0.15	0.304	
Model 2	Composite hip JPS absolute error (°)	Pain intensity (0–10 NRS)	0.18	0.13	0.18	−0.08 to 0.44	0.164	

Multiple linear regression analyses were performed to identify independent predictors of (Model 1) posturographic balance impairment (center-of-pressure sway velocity, eyes closed) and (Model 2) hip joint position sense (JPS) absolute error. Unstandardized coefficients (B), standard errors (SE), standardized coefficients (*β*), 95% confidence intervals (CI), and *p*-values are reported. All models were adjusted for age, body mass index (BMI), and pain intensity (numeric rating scale). Model fit is presented using R^2^ and adjusted R^2^ values. Statistical significance was set at *p* < 0.05.

B, unstandardized regression coefficient; BMI, body mass index; CI, confidence interval; COP, center of pressure; JPS, joint position sense; NRS, numeric rating scale; PPT, pressure pain threshold; SE, standard error; *β*, standardized regression coefficient; R^2^, coefficient of determination.

## 4 Discussion

The present study aimed to investigate hip proprioceptive accuracy in individuals with early hip osteoarthritis and to examine its associations with posturographic balance performance and pressure pain sensitivity. The findings demonstrated that individuals with early hip osteoarthritis exhibited clear impairments in hip joint position sense, reflected by lower accuracy, increased variability, and directional bias during repositioning tasks. These proprioceptive deficits were accompanied by consistent alterations in postural control, with higher instability observed across both simple and more challenging balance conditions. In addition, pressure pain thresholds were reduced at both local and remote sites, indicating heightened pain sensitivity. Importantly, proprioceptive impairments showed moderate associations with balance deficits, particularly under conditions with reduced visual input, suggesting a reliance on somatosensory function for postural control. Furthermore, inverse relationships between pressure pain sensitivity and proprioceptive accuracy indicated that increased pain sensitivity was linked to poorer sensorimotor performance. Regression analyses further supported these findings, identifying proprioceptive error and local pain sensitivity as independent contributors to balance impairment, and showing that both pain sensitivity and balance performance were significant determinants of proprioceptive deficits. Collectively, these results highlight the interconnected nature of sensorimotor dysfunction, postural instability, and altered pain processing in early hip osteoarthritis.

Impairments in hip joint position sense observed in individuals with early hip osteoarthritis can be attributed to both peripheral mechanoreceptor dysfunction and altered neuromuscular control (30). Degenerative changes within the hip joint, even at early stages, are known to affect capsuloligamentous and periarticular mechanoreceptors, thereby reducing afferent feedback necessary for accurate joint position sensing (18). This mechanism is supported by Alkhamis et al. (5), who reported compromised proprioceptive acuity in populations with hip and knee osteoarthritis due to structural alterations of the joints, and by Ahmad et al. (15), who demonstrated that early degenerative changes are sufficient to disrupt sensory input and motor coordination. In addition, impaired muscle activation patterns, particularly involving the hip stabilizers, may contribute to increased variability and directional error during repositioning tasks (31). Liu et al. (32) highlighted that altered muscle recruitment strategies in hip pathology affect dynamic joint control, while Kandakurti et al. (33) emphasized that proprioceptive deficits are closely linked to reduced sensorimotor integration in lower-limb disorders. Together, these mechanisms explain the observed reductions in accuracy and consistency of hip joint position sense in early hip osteoarthritis (18).

The observed associations between proprioceptive deficits, posturographic instability, and increased pain sensitivity reflect an interaction between sensorimotor dysfunction and altered nociceptive processing (4). Reduced proprioceptive input likely compromises postural control, particularly when visual feedback is limited, leading to higher reliance on impaired somatosensory pathways (34). This relationship is consistent with findings by Zeng et al. (35), who demonstrated that deficits in joint position sense are associated with higher postural sway in individuals with lower-limb osteoarthritis, and by Sarvestani et al. (36), who identified proprioception as a key determinant of balance performance. Furthermore, increased pain sensitivity, as indicated by reduced pressure pain thresholds, may interfere with central processing of sensory information, thereby exacerbating motor control deficits (37). Poesl et al. (38) reported that heightened nociceptive input can disrupt proprioceptive acuity and postural stability, while Dahmani et al. (39) provided evidence that central sensitization contributes to widespread sensory and motor alterations in osteoarthritis. The independent contributions of proprioceptive error and pain sensitivity to balance impairment, as well as their reciprocal relationship, underscore the role of both peripheral and central mechanisms in shaping functional deficits in early hip osteoarthritis (40).

### 4.1 Clinical significance

The findings of this study have direct clinical relevance for the early identification and management of hip osteoarthritis by highlighting measurable proprioceptive deficits, postural instability, and altered pain sensitivity even in the earliest disease stages. The use of clinically feasible tools such as digital inclinometers, posturography, and pressure pain threshold assessment provides a practical framework for comprehensive sensorimotor evaluation in routine practice. The demonstrated associations between impaired hip joint position sense and balance dysfunction, particularly under reduced sensory conditions, underscore the importance of incorporating proprioceptive and balance training into rehabilitation programs. Furthermore, the link between increased pain sensitivity and sensorimotor impairment suggests that interventions targeting both peripheral and central pain mechanisms may be necessary to optimize functional outcomes. These findings support a multidimensional clinical approach that integrates sensorimotor assessment with pain profiling to improve early-stage management strategies in early hip osteoarthritis.

### 4.2 Limitations

Several limitations should be considered when interpreting the findings. The cross-sectional design precludes causal inference regarding the relationships among proprioception, balance, and pain sensitivity. The study was limited to individuals with early hip osteoarthritis, which may restrict generalizability to more advanced stages or other hip pathologies. Although objective and reliable tools were used, potential measurement variability associated with digital inclinometry and posturography cannot be entirely excluded. Additionally, the absence of imaging-based assessments of muscle quality or neuromuscular activation limits the ability to fully explain the underlying mechanisms of sensorimotor impairment. The classification of early hip osteoarthritis was also based primarily on symptoms and Kellgren–Lawrence grade 1–2 radiographic findings. Although this approach is widely used in early osteoarthritis research, it may not fully capture the heterogeneity of early disease presentation, as radiographic changes do not always correspond with structural pathology, functional impairment, or symptom severity. Future studies should consider incorporating additional clinical, functional, and imaging-based indicators to improve the characterization of early hip osteoarthritis. In addition, no formal adjustment for multiple comparisons was applied because the study was exploratory in nature and involved several between-group and correlational analyses. Consequently, the findings should be interpreted with caution, as the possibility of an increased Type I error rate cannot be excluded. Future research should employ longitudinal designs to determine temporal relationships and causal pathways, and interventional studies are needed to evaluate whether targeted proprioceptive and pain-modulation therapies can improve balance and functional outcomes. Incorporating advanced biomechanical analyses, neuromuscular assessments, and central sensitization measures would further enhance understanding of the multifactorial nature of dysfunction in early hip osteoarthritis.

## 5 Conclusion

Early hip osteoarthritis is characterized by significant impairments in hip joint position sense, reduced postural stability, and increased pain sensitivity at both local and remote sites. Proprioceptive deficits are moderately associated with balance impairments, particularly under conditions of reduced visual input, and are inversely related to pressure pain thresholds. Both proprioceptive error and local pain sensitivity independently contribute to postural instability, while pain sensitivity and balance performance are significant determinants of proprioceptive accuracy. These findings demonstrate a clear interaction between sensorimotor dysfunction and altered pain processing in early hip osteoarthritis, emphasizing the importance of integrated assessment approaches targeting both domains.

## Data Availability

The datasets generated and/or analyzed during the current study are publicly available in the Zenodo repository and can be accessed via the following DOI: Zenodo Dataset (DOI: 10.5281/zenodo.10598373) https://zenodo.org/records/10598373.
